# Comparative Transcriptomics of Root Development in Wild and Cultivated Carrots

**DOI:** 10.3390/genes9090431

**Published:** 2018-08-24

**Authors:** Gabriela Machaj, Hamed Bostan, Alicja Macko-Podgórni, Massimo Iorizzo, Dariusz Grzebelus

**Affiliations:** 1 Institute of Plant Biology and Biotechnology, Faculty of Biotechnology and Horticulture, University of Agriculture in Krakow, 31425 Krakow, Poland; gmachaj@ogr.ur.krakow.pl (G.M.); a.macko@urk.edu.pl (A.M.-P.); 2 Plants for Human Health Institute, Department of Horticultural Science, North Carolina State University, Kannapolis, NC 28081, USA; hbostan@ncsu.edu (H.B.); miorizz@ncsu.edu (M.I.)

**Keywords:** *Daucus carota* L., DEGs, domestication, gene expression regulation, storage root, transcriptome

## Abstract

The carrot is the most popular root vegetable worldwide. The genetic makeup underlying the development of the edible storage root are fragmentary. Here, we report the first comparative transcriptome analysis between wild and cultivated carrot roots at multiple developmental stages. Overall, 3285, 4637, and 570 genes were differentially expressed in the cultivated carrot in comparisons made for young plants versus developing roots, young plants versus mature roots, and developing roots versus mature roots, respectively. Of those, 1916, 2645, and 475, respectively, were retained after filtering out genes showing similar profiles of expression in the wild carrot. They were assumed to be of special interest with respect to the development of the storage root. Among them, transcription factors and genes encoding proteins involved in post-translational modifications (signal transduction and ubiquitination) were mostly upregulated, while those involved in redox signaling were mostly downregulated. Also, genes encoding proteins regulating cell cycle, involved in cell divisions, development of vascular tissue, water transport, and sugar metabolism were enriched in the upregulated clusters. Genes encoding components of photosystem I and II, together with genes involved in carotenoid biosynthesis, were upregulated in the cultivated roots, as opposed to the wild roots; however, they were largely downregulated in the mature storage root, as compared with the young and developing root. The experiment produced robust resources for future investigations on the regulation of storage root formation in carrot and *Apiaceae*.

## 1. Introduction

The carrot (*Daucus carota* subsp. *sativus* L.) is one of the most important vegetable crops in the world with a current annual yield of more than 40 million tons produced on ca. 1.2 million hectares [1]. The orange-rooted carrot owes its popularity to its high nutritional, palatable roots, which are the main source of beta-carotene (provitamin A) in the human diet [2]. The cultivated carrot (2n = 2x = 18, haploid genome size of 473 Mb) was derived from the wild carrot (*Daucus carota* L. subsp. *carota*) ca. 1100 years ago in Central Asia [3]. The first domesticated carrots were purple and yellow [4]. Carrots producing orange roots likely appeared in the Netherlands in the 16th century [5] and spread to other countries, becoming predominant in the commercial production. Essential differences between the cultivated carrot and its wild progenitor, including the ability to form fleshy roots, minimal lateral root branching, strictly biennial growth habit, and elevated sugar content in the roots, define the carrot domestication syndrome [6]. Wild carrot roots, as opposed to those produced by cultivated varieties, are white, woody, and branched, whereas domesticated carrots have pigmented, smooth, fleshy roots with nice flavor [7].

A previous study suggested hormonal control of storage root development [8]. Rong et al. (2014) reported on genes probably associated with the storage root formation (e.g., those encoding water channel proteins) and emphasized that changes in gene expression might be essential for carrot domestication [7]. The recent release of a high-quality carrot genome assembly [9] allows for more systematic research to identify the genetic factors responsible for the transition from the thin and woody root of the wild carrot to the fleshy storage root of the cultivated carrot.

Here, we report on the first high coverage comparative transcriptome analysis of wild and cultivated carrot accessions in the course of root development. We analyzed transcriptomes of a cultivated breeding line 2874B producing orange roots and a wild *D*. *carota* subsp. *commutatus* in three biological replicates in three time points. We identified and compared differentially expressed genes (DEGs) during root development in cultivated and wild carrots. Our comprehensive transcriptomic analyses provide insight into the genetic complexity and expand our knowledge about the genetic basis of storage root development.

## 2. Materials and Methods

### 2.1. Plant Materials

**Figure 1:**
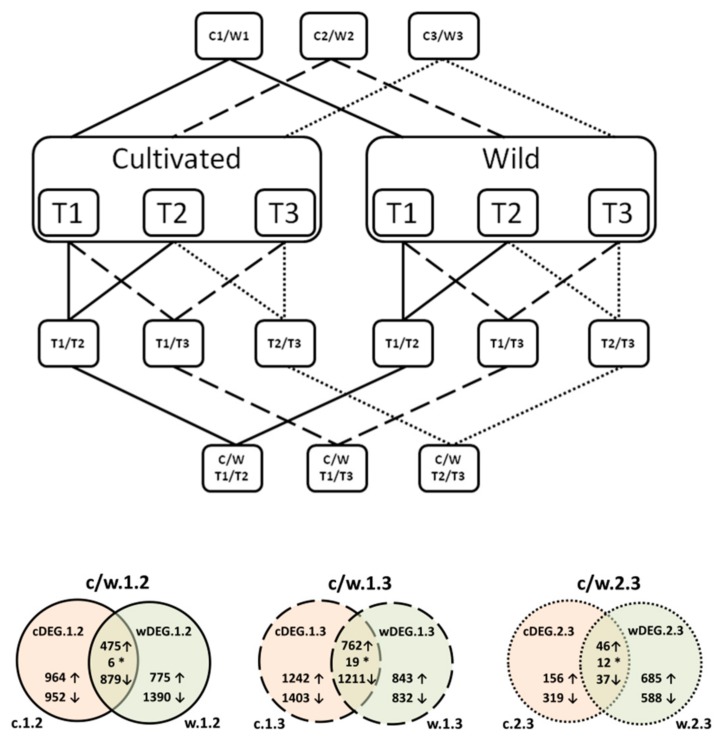
A diagram illustrating the applied experimental strategy and Venn diagrams showing numbers of differentially expressed genes (DEGs) identified in comparisons between wild (w) and cultivated (c) carrots in each of the three timepoints. ↑—upregulated genes; ↓—downregulated genes; *—genes with discordant expression patterns between wild and cultivated carrots.

An orange-rooted cultivated breeding line 2874B and a wild accession of *D*. *carota* subsp. *commutatus* (JKI-W232/07) were grown in pots in the greenhouse. Samples for RNA extraction were collected at three timepoints (i.e., T1—55 days after sowing (young plants with two true leaves); T2—110 days after sowing (developing roots); and T3—165 days after sowing (mature roots)). At each timepoint, three randomly selected plants per accession were harvested, resulting in three biological replicates per timepoint and the total of 18 samples used for transcriptome sequencing (Figure 1, Table S1 in the Supplementary Materials).

### 2.2. RNA Extraction and RNAseq

Total RNA was extracted from fresh plantlets (T1) and roots (T2, T3) using NucleoSpin RNA (Macherey-Nagel, Düren, Germany) combined with TRIzol Reagent (Thermo Fisher Scientific, Waltham, MA, USA; Invitrogen, Carlsbad, CA, USA) and Fruit-mate for RNA Purification reagent (Clontech; Takara, Otsu, Japan) as described by the manufacturers. DNA contaminations were removed with the Turbo DNA-free kit (Thermo Fisher Scientific; Ambion; Austin, TX, USA) following the manufacturer’s protocol. The quality and quantity of RNA was determined using NanoDrop 2000c (Thermo Fisher Scientific) and gel electrophoresis. mRNA was obtained from RNA samples using NEBNext^®^ Poly(A) mRNA Magnetic Isolation Module (NEB; Herts, UK). After quality control (Agilent 2100 Bioanalyzer; Agilent Technologies, Palo Alto, CA, USA) and library preparation (NEBNext^®^ Ultra™ Directional RNA Library Prep Kit for Illumina, NEB), cDNA was sequenced in PE100 (paired ends mode, 100 bp) using HighSeq 4000 (Illumina; San Diego, CA, USA) next generation sequencing platform. 

### 2.3. RNAseq Data Analysis

The raw sequences were subjected to adaptor removal and quality control using Trimmomatic [10] using the following parameters: Leading: 20; Tailing: 20; SlideWindow: 5:20; MinLen: 50; TRueSeq3-PE; and fa.2:30:10. Bowtie 2 [11] was used to map the high-quality reads to the carrot rRNA sequences extracted from the reference genome annotation files [9] (NCBI accession LNRQ01000000) (parameters: fast end-to-end mode; D: 10; R: 2; N: 0; L: 22; and i: S,0,2.50). Reads matching the rRNA sequences were discarded from further analysis. Reads were then mapped to the carrot DH1 reference genome [9] (NCBI accession LNRQ01000000) using a 2-pass alignment approach with STAR [12] with the following parameters: outSAMmapqUnique: 50; outFilterMultimapNmax: 20; alignSJoverhangMin: 8; alignSJDBoverhangMin: 1; outFilterMismatchNmax: 999; outFilterMismatchNoverLmax: 0.04; alignIntronMin: 20; alignIntronMax: 1,000,000; and alignMatesGapMax: 1,000,000. De novo isoforms and transcripts were identified with StringTie [13] using the following parameters: min-length: 200; junction-coverage: 10; and min-coverage: 10. The reference annotation was merged with new isoform data using Cuffmerge [14]. Candidate coding regions within transcripts were identified with TransDecoder [15].

Kallisto [16] was used to estimate expression levels in each sample separately. Read counts were expressed in TPM (transcripts per million) units. Differentially expressed genes were identified using three R packages: DESeq2 [17], EBSeq [18], and edgeR [19]. DEGs (minimum read count = 10) were called when false discovery rate (FDR) ≤0.05 was reported for all three algorithms (Figures S1–S3 in the Supplementary Materials). First, we compared expression patterns in T1 versus T2, T1 versus T3, and T2 versus T3, separately for the cultivated (c) and the wild (w) accessions (Figure 1). Genes with expression changes validated as significant by all three DEG detection methods were retained, resulting in the six datasets (c.1.2; c.1.3; c.2.3; w.1.2; w.1.3; and w.2.3) from these pairwise comparisons (Figure 1). Finally, to identify genes essential for the storage root development, we compared results obtained for the cultivated and the wild accessions in the following manner: c.1.2 versus w.1.2 (c/w.1.2); c.1.3 versus w.1.3 (c/w.1.3); and c.2.3 versus w.2.3 (c/w.2.3). Further, genes differentially expressed in cultivated and wild carrots were retained, resulting in six datasets (cDEG.1.2; cDEG.1.3; cDEG.2.3, wDEG.1.2; wDEG.1.3; and wDEG.2.3) comprising DEGs likely engaged in the root development (Figure 1). In addition, we identified DEGs differentiating the wild and the cultivated accessions in each timepoint (i.e., T1, T2, and T3), resulting in three additional datasets wcDEG.1, wcDEG.2, and wcDEG.3. The detailed explanation of the numerical code system used for reporting the data is presented in Table 1. DEGs from each comparison were used to perform co-expression clustering and gene ontology (GO) enrichment/association analysis. To identify the genes with similar pattern of expression in each comparison, *k*-means clustering [20,21] was carried out using *k* as the number of clusters defined for each set depending on the sample size (Tables S11–S19 in the Supplementary Materials) using the GO annotation of carrot genes v2.0 [9]. GO enrichment analysis was conducted on each cluster of each comparison independently using Fisher exact test considering FDR ≤0.05 (Tables S20 and S21 in the Supplementary Materials). All reported gene functional annotations are reported according to Iorizzo et al. (2016) [9].

## 3. Results and Discussion

### 3.1. RNAseq Summary and Identification of Novel Transcripts

We obtained ca. 18,400,000 read pairs per sample, more than 90% (71.9–97.7%, mean = 93%; Table S2 in the Supplementary Materials) were uniquely mapped to the reference carrot genome (LNRQ01000000) and used to identify DEGs and novel transcripts. We identified 9978 novel transcripts, comprising 7765 novel isoforms of annotated genes, 567 novel intergenic transcripts, 510 and 773 exon-overlapping transcripts (sense and antisense, respectively), and 339 novel transcripts in intronic regions (Table S3 in the Supplementary Materials).

### 3.2. Differential Expression

**Figure 2:**
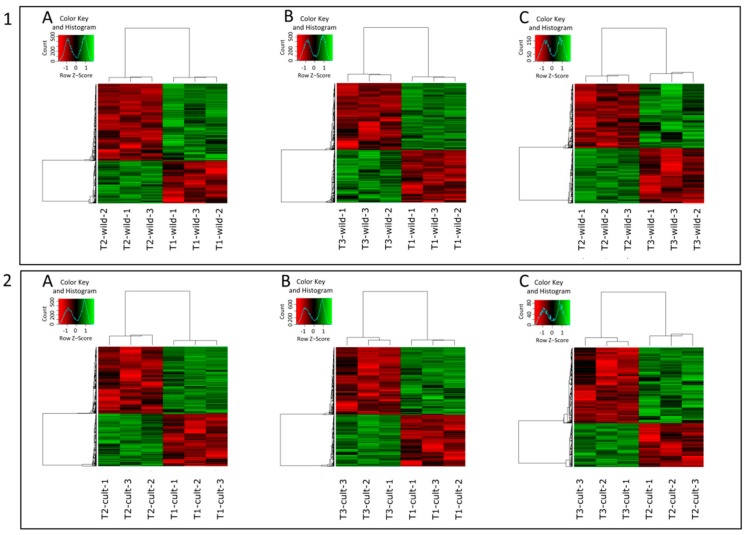
Heatmaps representing DEGs in wild (**1**) and cultivated (**2**) carrots for time points: T1 vs. T2 (**A**), T1 vs. T3 (**B**), and T2 vs. T3 (**C**). Row z-score represents normalized expression of DEGs.

DEGs were identified in all possible timepoint pairwise comparisons, separately for the wild (w.1.2, w.1.3, and w.2.3) and the cultivated (c.1.2, c.1.3, and c.2.3) carrot accessions (Figure 2).

**Figure 3:**
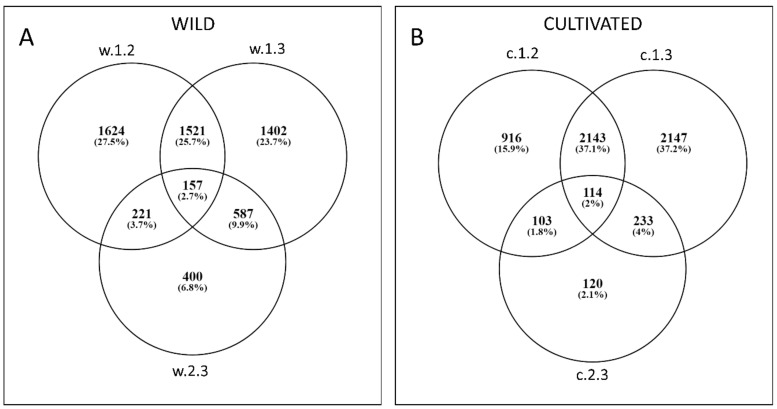
Venn diagrams showing number of DEGs in wild (**A**) and cultivated (**B**) carrots for each comparison (T1 vs. T2, T1 vs. T3, and T2 vs. T3).

In the wild carrot, a total of 5930 genes were differentially expressed in at least one comparison (Figure 2(1)). Among them, 157 genes were differentially expressed in all three comparisons (Figure 3A). 3525 DEGs were identified in w.1.2 (Figure 2(1A) and 3A; Table S4 in the Supplemental Materials) with 2272 and 1253 being down- and upregulated, respectively. In w.1.3, 3667 DEGs were identified, with 2052 and 1615 down- and upregulated, respectively (Figure 2(1B) and 3A; Table S5 in the Supplemental Materials). w.2.3 resulted in 1368 DEGs, of which 737 and 631 were down- and upregulated, respectively, in the mature root of the wild accession (Figure 2(1C) and 3A; Table S6 in the Supplemental Materials).

5776 genes were differentially expressed in the cultivated carrot (Figure 2(2)), of which 114 were differentially expressed in all three comparisons (Figure 3B). In c.1.2, 1442 and 1843 DEGs were up- and downregulated, respectively (Figure 2(2A) and 3B; Table S7 in the Supplemental Materials). In c.1.3, 4637 DEGs were identified, of which 2013 were upregulated and 2626 were downregulated (Figure 2(2B) and 3B; Table S8 in the Supplemental Materials). In c.2.3, 570 genes were differentially expressed, comprising 208 and 362 up- and downregulated, respectively (Figure 2(2C) and 3B; Table S9 in the Supplemental Materials).

We then partitioned the sets of DEGs into three categories: (1) those regulated in a similar fashion during the development of wild and cultivated roots (c/w.1.2; c/w.1.3; and c/w.2.3, with 1360; 1992; and 95 genes, respectively); (2) those specific to the cultivated carrot (cDEG.1.2; cDEG.1.3; and cDEG.2.3, comprising 1916; 2645; and 475 genes, respectively) and (3) those specific to the wild carrot (wDEG.1.2; wDEG.1.3; and wDEG.2.3, comprising 2165; 1675; and 1272 genes, respectively) (Figure 1). The data on the expression level and profile cluster, GO association, and enrichment for each of the above DEG sets in each comparison are provided in the Supplementary Materials (Tables S11–S21). A very small fraction of genes (6, 19, and 12 genes for c/w.1.2; c/w.1.3; and c/w.2.3, respectively) differentially regulated in both wild and cultivated carrots, but showing discordant expression patterns, was revealed (Figure 1, Supplementary Tables S10–S13). However, in order to investigate genome-wide relationships and identify groups of genes involved in the formation of the carrot storage root, in the following section, we focused on the DEG sets specific to the root of the cultivated carrot (category 2 listed above).

#### 3.2.1. cDEG.1.2: Genes Differentially Expressed in the Developing Storage Root (T2), as Compared with the Young Plant (T1)

**Figure 4:**
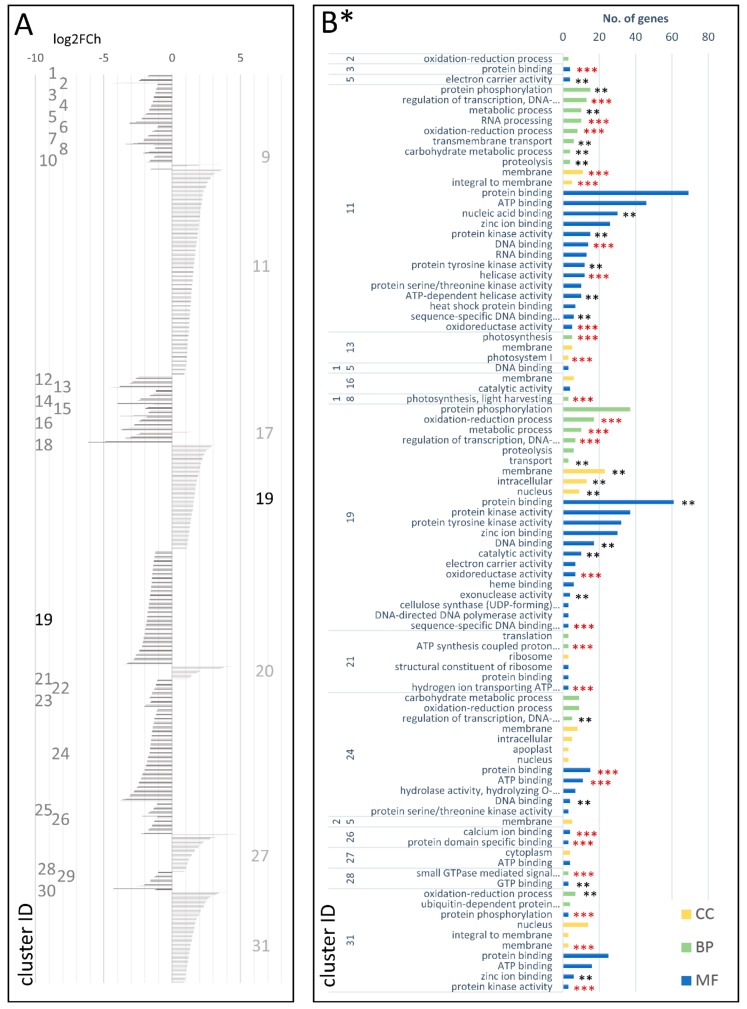
Clusters of DEGs in the cultivated carrot (young plant vs. developing root; cDEG.1.2) showing log2FoldChange values (**A**) and GO enrichment results (**B**). CC—cellular component; BP—biological process MF—molecular function; *—number of genes in set >2; **—adjusted *p*-value < 0.01; and ***—adjusted *p*-value < 0.001.

DEGs attributed to the cDEG.1.2 dataset were grouped into 31 co-expressed gene clusters (Figure 4A, Table S14 of the Supplementary Materials). Of those, six and 24 clusters comprised only up- and downregulated DEGs, respectively, while the largest cluster, cDEG.1.2.19, included 222 up- and 246 downregulated DEGs. GO-enriched categories with regard to biological processes (BP) comprised protein phosphorylation, oxidation-reduction process, and regulation of transcription. Protein and ATP binding were the most enriched molecular functions (MF), while membrane and nucleus were the most enriched cellular components (CC) (Figure 4B; Table S20 in the Supplementary Materials).

A more detailed functional analysis of genes comprising the up-regulated clusters pointed at their possible significance in the process of storage root development. For example, cluster cDEG.1.2.11 grouping 433 DEGs (log2FCh = 0.88–3.70) included genes involved in transcription regulation, prevention of vegetative-to-reproductive phase switch (flowering time negative regulation), root development, mitotic cell division, mRNA splicing, and translation. In cDEG.1.2.20, 29 DEGs (log2FCh = 1.13–2.27) were attributed to multicellular organism development, transcription, and light-dependent circadian clock regulation, while cDEG.1.2.27 included 80 DEGs (log2FCh = 0.89–4.67) related to cell division/proliferation, plant growth and development, circadian clock, cell wall biogenesis, and transcription regulation. In cluster cDEG.1.2.31, 192 DEGs (log2FCh = 0.89–3.95) were involved in root development, cell division, vascular strand development, limitation of programmed cell death, hormonal response, sugar transport, and transcription regulation.

DEGs grouped in the 24 clusters downregulated in the cultivated but not in the wild developing root, as compared with the young plant stage, were enriched in genes associated with oxidation-reduction process, carbohydrate metabolic process, regulation of transcription, nucleosome assembly, ATP synthesis coupled proton transport, and photosynthesis. In terms of molecular function, they were attributed to ATP binding, DNA binding, hydrolase activity, hydrolyzing O-glycosyl compounds, hydrogen ion transporting ATP synthase activity, and protein domain specific binding (Figure 4B; Table S20 in the Supplementary Materials).

#### 3.2.2. cDEG.1.3—Genes Differentially Expressed in the Mature Storage Root (T3), as Compared with the Young Plant (T1)

**Figure 5:**
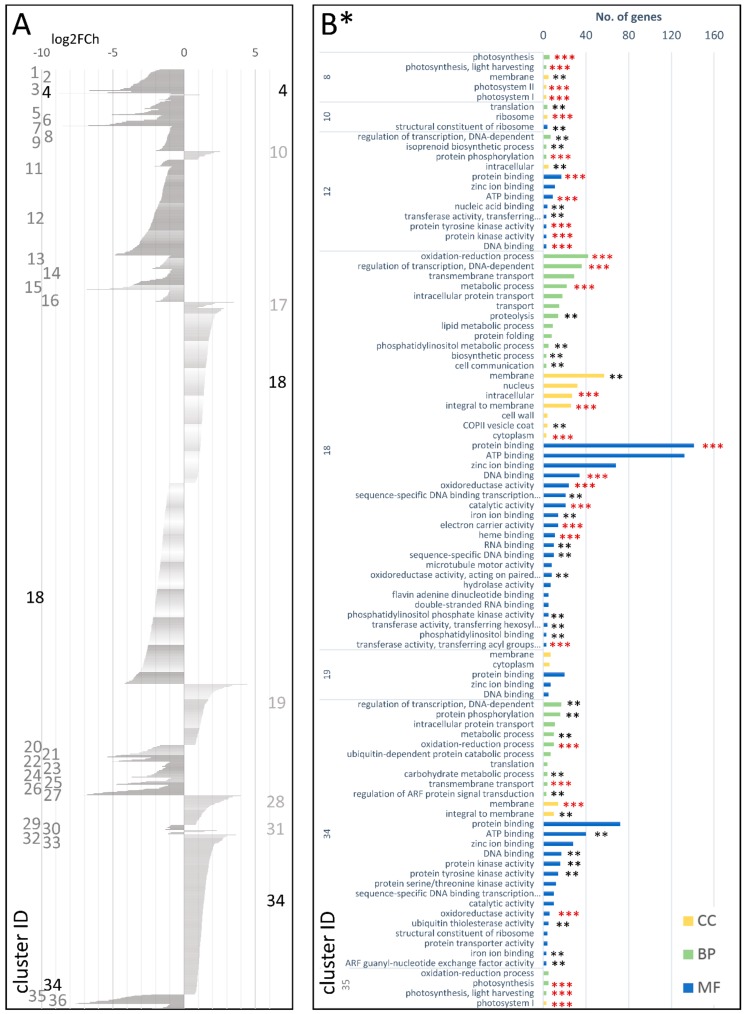
Clusters of DEGs in the cultivated carrot (young plant v. mature root; cDEG.1.3) showing log2FoldChange values (**A**) and GO enrichment results (**B**). Several clusters and enrichment terms in (**B**) were omitted for the clarity of presentation. CC—cellular component; BP—biological process MF—molecular function; *—number of genes in set >2; **—adjusted *p*-value < 0.01; and ***—adjusted *p*-value < 0.001.

In total, 2654 DEGs were identified in the cDEG.1.3 set, of which 1242 and 1403 were up- and downregulated in the mature carrot storage root, respectively. They were grouped into 36 co-expressed clusters (Figure 5A; Table S15 in the Supplementary Materials), of which six were upregulated and 29 downregulated and one large cluster (cDEG.1.3.18) comprised both up- and down-regulated genes (492 and 568, respectively). The up-regulated clusters in cDEG.1.3 were enriched in genes involved in regulation of transcription, oxidation-reduction process protein phosphorylation, transmembrane transport, and carbohydrate metabolism (biological processes, BPs) and protein/ATP/zinc ion/DNA binding (molecular functions, MFs) and were mostly localized in the membrane or the nucleus (cellular components, CCs) (Figure 5B; Table S20 in the Supplementary Materials). More detailed functions could have been attributed to upregulated DEGs grouped in individual clusters (e.g., cell wall biogenesis, plant/root development, and translation (cDEG.1.3.10; log2FCh = 0.91–2.84); plant development, cell proliferation, and hormonal regulation (cDEG.1.3.17; log2FCh = 0.96–4.32); transcription, cell wall biogenesis, hormonal signaling, and lateral root formation (cDEG.1.3.19; log2FCh = 0.79–4.40); regulation of transcription, plant development, hormonal signaling, cell wall organization, and xylogenesis in roots (cDEG.1.3.28; log2FCh = 0.79–4.40); cell wall biogenesis and water transport (cDEG.1.3.31; log2FCh = 1.42–3.11); regulation of transcription, plant development, cell growth, cell wall biogenesis, maintenance of the vegetative phase, mRNA splicing, translation, ubiquitin-dependent protein catabolic process, protein transport, and vesicle-mediated transport (cDEG.1.3.34; log2FCh = 0.77–3.63)).

The 29 downregulated clusters in cDEG.1.3 comprised genes associated with photosynthesis and light harvesting, nucleosome assembly, regulation of transcription, oxidation-reduction process, and isoprenoid biosynthesis. Many genes from these clusters were components of membrane, nucleosome, oxygen evolving complex, and photosystems I and II (Figure 5B; Table S20 in the Supplementary Materials).

#### 3.2.3. cDEG.2.3—Genes Differentially Expressed in the Mature Storage Root (T3), as Compared with the Developing Storage Root (T2)

**Figure 6:**
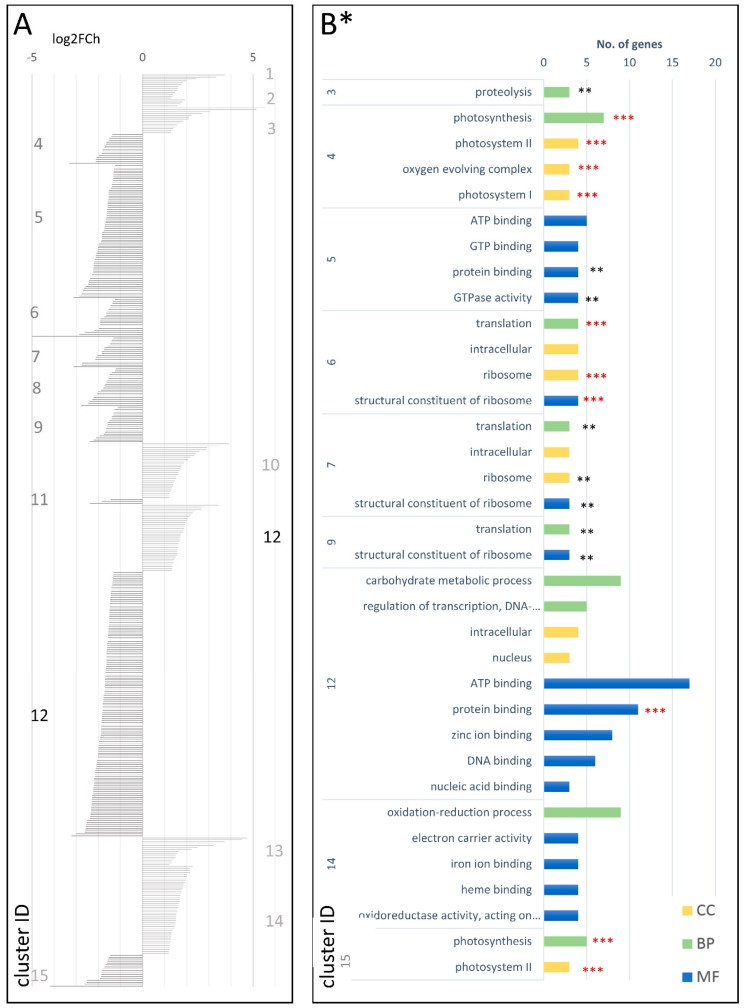
Clusters of DEGs in cultivated carrot (developing root vs. mature root; cDEG.2.3) showing log2FoldChange values (**A**) and GO enrichment results (**B**). CC—cellular component; BP—biological process; MF—molecular function; *—number of genes in set >2; **—adjusted *p*-value < 0.01; and ***—adjusted *p*-value < 0.001.

A much less numerous set of DEGs was identified in cDEG.2.3, including the total of 475 genes, of which 156 and 319 were up- and downregulated, respectively. They were grouped into 15 co-expressed clusters (Figure 6A; Table S16 in the Supplementary Materials) comprising six and eight clusters up- and downregulated in the mature root, respectively. One cluster (cDEG.2.3.12) comprised 35 upregulated and 138 downregulated DEGs. With respect to the biological processes, the upregulated genes were enriched in those involved in DNA-dependent regulation of transcription, oligopeptide transport, and protein phosphorylation, while the most common biological functions were protein binding, ATP binding, and transport. Nuclear and intracellular localizations prevailed. The downregulated gene clusters comprised DEGs related to oxidation-reduction process, translation, and photosynthesis (BP), ATP binding (MF), and membrane, ribosomes, and photosystems I and II (CC) (Figure 6B; Table S20 in the Supplementary Materials).

A more detailed analysis of individual clusters showed that cDEG.2.3.2 contained four upregulated genes (log2FCh = 1.57–1.92) associated with water uptake and transport and that cDEG.2.3.1 grouped 13 upregulated genes (log2FCh = 1.28–3.71) likely involved in root/lateral root development and water transport. In the clusters cDEG.2.3.3 (14; log2FCh = 1.28–5.56) and cDEG.2.3.13 (15; log2FCh = 1.21–4.72), upregulated DEGs were associated with cell wall formation/modification, phloem/xylem histogenesis, and cell differentiation. Genes from cluster cDEG.2.3.10 (29; log2FCh = 1.20–3.90) could have been attributed to sugar/carbohydrate metabolism and transport, flavonoid biosynthesis, and transcriptional regulation of plant development. Also, the cluster cDEG.2.3.14 comprised upregulated genes (log2FCh = 1.18–2.27) probably related to cell wall organization/biosynthesis, regulation of transcription, and carbohydrate metabolism. Probable functions of upregulated genes in the cluster cDEG.2.3.12 (log2FCh = 1.33–3.42) may be related to polysaccharide/sugar metabolism, hormonal regulation (e.g., auxin, abscisic acid (ABA)), root and vascular tissue development, and plant and cell growth.

The downregulated genes in the cDEG2.3 set were primarily involved in chlorophyll biosynthesis, photosynthesis (photosystem I and II), and chloroplast formation, but also with sugar transport and sucrose and starch metabolism (Figure 5B; Table S20 in the Supplementary Materials). Interestingly, two key genes involved in carotenoid biosynthesis (i.e., *PSY2* (phytoene synthase 2) and *LCYE* (lycopene epsilon cyclase)) were found to be significantly downregulated in clusters cDEG2.3.8 and cDEG2.3.5, respectively.

#### 3.2.4. DEGs between Wild in Cultivated *D. carota* at the Three Timepoints (wcDEG.1, wcDEG.2, and wcDEG.3)

**Figure 7:**
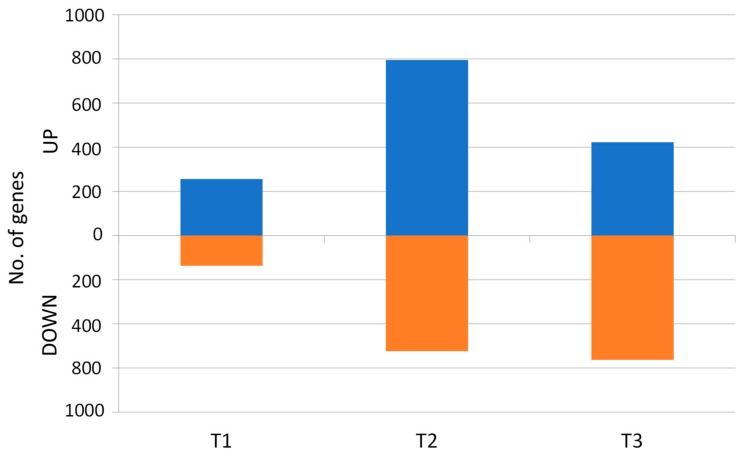
DEGs up- and downregulated (blue and orange bars, respectively) in the cultivated carrot at three timepoints, as related to the wild carrot.

In addition to the investigation on developmentally differentially regulated genes, we performed analysis aiming at the identification of DEGs in the two accessions directly differentiating the wild and the cultivated plants at each timepoint (Tables S22–S26 in the Supplementary Materials). In T1, 393 DEGs were identified, of which 256 and 137 were up- and downregulated, respectively. They were grouped into 14 clusters, of which 10 were upregulated in the cultivated, two were downregulated, and two comprised up- and downregulated genes (Table S22 in the Supplementary Materials). In T2, 1319 genes were differentially expressed; they were grouped into 26 clusters, of which 19 were upregulated and six were downregulated in the cultivated carrot. The one remaining cluster comprised both up- and downregulated genes (Table S23 in the Supplementary Materials). In T3, 986 DEGs were revealed, and 22 clusters were distinguished, of which only seven were upregulated in the mature storage roots, while 14 clusters included downregulated genes and one grouped both up- and downregulated DEGs (Table S24 in the Supplementary Materials). In summary, a general shift in gene expression could have been observed, from higher expression levels in the young plants and developing roots of the cultivated carrot to lower expression in the mature storage root, relative to the wild carrot (Figure 7). 

GO analysis revealed that DEGs resulting from the comparison between the wild and the cultivated accession could have been attributed to diverse biological process categories. We focused on GO categories that were enriched for the developmentally regulated DEGs. Genes involved in processes related to photosynthesis were systematically upregulated in the cultivated carrot at all timepoints (clusters wcDEG.1.3, wcDEG.1.6, wcDEG.1.12, wcDEG.2.1, wcDEG.2.12, wcDEG.2.19, wcDEG.2.20, wcDEG.2.21, wcDEG.2.26, wcDEG.3.7, and wcDEG.3.9). In contrast, DEGs involved in the regulation of transcription and protein phosphorylation were grouped into up- and downregulated clusters, mostly at T2 and T3 (e.g., wcDEG.3.3 and wcDEG.3.19 vs. wcDEG.2.10 and wcDEG.3.6, respectively), similar to those involved in the sucrose and carbohydrate metabolism (wcDEG.2.8 vs. wcDEG.2.6), while DEGs involved in the cell wall formation were upregulated in the cultivated carrot only at T1 (S22–S26 in the Supplementary Materials). This shows that the analysis performed on the developmentally regulated DEGs (cDEGs) provided more insight into the process of storage root formation than the direct comparison of transcriptomes of the two accessions (wcDEGs).

### 3.3. Genetic Determinants of the Carrot Storage Root Development

RNAseq has been previously used to elucidate expression changes underlying the development of storage organs (e.g., sweet potato [22]; radish [23]). Previous reports on carrot root transcriptomes [7,24] provided preliminary insight into mechanisms governing the storage root formation, while here we presented the first genome based, comparative transcriptome analysis of root development in wild and cultivated carrots. Using a replicated sampling at three developmental stages, we were able to recognize DEGs during root growth in both wild and edible carrots. Most importantly, utilizing wild carrot transcriptomes as a reference, we identified subsets of genes (cDEGs) likely essential for the development of fleshy roots in the cultivated carrots at different stages of root growth.

In the following subsections, using evidence from the analysis of the cDEG datasets, we show that storage root development requires a complex array of regulatory processes, including transcriptional regulation, post-translational protein modifications (phosphorylation, ubiquitination), redox, and hormonal signaling. They likely regulate a range of developmental processes (e.g., cell division and proliferation, developmental phase control, cell wall development, vascular tissue biogenesis, carbohydrate metabolisms, water uptake and transport).

#### 3.3.1. Regulation of the Carrot Storage Root Development

##### 3.3.1.1. Transcription Factors

In cultivated carrot roots we identified a number of differentially expressed transcription factors (TFs) of several families (i.e., bZIP, ERF, GATA, NF-Y, WRKY, bHLH, GTE, MYB1R1, TCP, and trihelix TFs being the most abundant (Table 2; Tables S14–S16 in the Supplementary Materials)). Interestingly, they were mostly upregulated at later stages of the root development in comparison with their expression in young plants. They were clustered primarily in cDEG.1.2.11, cDEG.1.2.19, and cDEG.1.2.27 (young plant vs. immature root); cDEG.1.3.19 and cDEG.1.3.34 (young plant vs. mature root); and cDEG.2.3.5 and cDEG.2.3.14 (immature vs. mature root). Several representatives of the abovementioned TF families have been reported as being involved in the regulation of root development (NF-Y [25,26]), organ shape (trihelix TF [27]), and root epidermal cell fate specification (EGL1 and bHLH [28]).

Moreover, we identified nine DEGs representing the AT-hook motif containing nuclear localized (AHL) family. AHLs act as transcription regulators due to their capability of binding AT-rich DNA fragments and have been reported as regulators of hypocotyl growth [29], defense response [30], and flowering [31]. Notably, AHL28-like (LOC108210904) showed a very high change in its level of expression (log2FCh = 3.3 and 1.689) in two comparisons (cDEG.1.3 and cDEG.2.3, respectively). Also, AHL5-like (LOC108209748) was found among differentially expressed AHLs. Previously, it has been proposed as a candidate domestication gene in carrot (*DcAHLc1*), likely involved in the storage root development [6], as it was associated with a region under strong selection in the cultivated carrot [32]. Here, *DcAHLc1* gene was significantly downregulated (FDR ≤ 0.001 and 0.01) in both cultivated (T1 vs. T3: log2FCh = −1.830) and wild (T1 vs. T3: log2FCh = −1.922; T2 vs. T3: log2FCh = −1.443) carrots. Possibly, the fact that DcAHLc1 is regulated in a similar fashion during root growth both in cultivated and wild carrots supports the view proposed by Macko-Podgórni et al. (2017) that structural differences of the DcAHLc1 variant present in cultivated carrots determine the effect of the gene on the development of storage roots [6].

##### 3.3.1.2. Post-Translational Protein Modifications

Protein modifications such as (de)phosphorylation, sumoylation, and ubiquitination may affect plant metabolism, growth, and development [33,34,35]. We identified DEGs during storage root growth encoding proteins involved in post-translational modifications.

Several protein kinases were found to be differentially regulated during the development of the carrot storage root. Upon comparison of expression profiles between the young plant and the immature root, most of them were assigned to upregulated clusters cDEG.1.2.11 and cDEG.1.2.19. Also, in the young plants versus the mature roots protein kinases were upregulated, most of them clustering within cDEG.1.3.34. The co-expression of protein kinases and genes involved in the plant development implies that they are important factors controlling the storage root formation. 

We also identified a number of DEGs involved in protein ubiquitination. Most of them were upregulated only in storage roots but silenced or not differentially expressed in the roots of wild carrots. Among them, genes associated with CUL4-DDB1 ubiquitin E3-ligase complex formation were most abundant in the cDEG datasets, while none of them were differentially expressed in the wild versus cultivated comparisons (wcDEG datasets), indicating that they were developmentally regulated in the roots of the cultivated carrot. They clustered within cDEG.1.2.11 and cDEG.1.2.19 and cDEG.1.3.18 and cDEG.1.3.34. Those clusters were mainly associated with root development, cell wall development, phloem/xylem formation, and flowering time regulation. CUL4-DDB1 ubiquitin E3-ligase complex regulates proteolysis of key proteins in transcription, replication, and DNA repair [36]. It has been reported to be essential for plant development [37].

Sumoylation genes have been described as plant growth and drought stress regulators [38]. Differentially expressed *SIZ1* genes in carrots were clustered within cDEG.1.2.11; cDEG.1.2.19; and cDEG.1.3.18. These clusters were associated with root development and mitotic cell divisions.

##### 3.3.1.3. Hormonal Signaling

Control of the plant development by hormones as main regulators has been widely studied. Auxins, cytokinins, gibberellins, ethylene, abscisic acid, and brassinosteroids have been reported as essential factors in both promotion and inhibition of plant growth in many species [39]. Differential expression of dozens of hormone-related genes during carrot storage root growth has been previously reported by Wang et al. (2015), who showed that plant hormones regulate carrot root growth in a stage-dependent manner [8]. We also identified several DEGs (e.g., abscisic acid receptor PYL4-like—LOC108215964; auxin efflux carrier component 4-like—LOC108196170; auxin response factor 18-like—LOC108223505; auxin transport protein BIG—LOC108213127; gibberellin-regulated protein 6-like—LOC108210736; and GTP-binding protein BRASSINAZOLE INSENSITIVE PALE GREEN 2—LOC108227738) involved in hormonal signaling. These genes were differentially expressed only in storage roots and not in wild carrot roots. While none of these genes showed drastically different expression levels throughout the whole period of root development, two of them (LOC108223505 and LOC108227738) were upregulated in cultivated roots, as compared with wild roots, at T2, the former one still being up-regulated at T3 (Tables S23 and S24 in the Supplementary Materials). Possibly, alterations of the hormonal control are important elements of the storage root growth regulation.

##### 3.3.1.4. Redox Signaling

Redox regulation has been reported as a main factor (next to phytohormonal signaling) controlling cell cycle, and plant growth and development [40], as well as biosynthesis of biological compounds (e.g., carotenoids [41]). Redox regulation affects nearly every stage of plant and root development, from breaking ABA-induced seed dormancy to the development of root meristem, lateral roots, and root hairs [40]. Oxidoreductase activity was one of the most enriched GO terms among all DEGs in the storage root. For example, only in cDEG.1.3.18 more than 20 genes were associated with the oxido-reduction process. Generally, most of them were downregulated during the growth of storage roots.

##### 3.3.1.5. Regulation Complexity

As shown above, the regulation of the carrot storage root development involves several interconnected mechanisms. TF activity can be regulated by plant hormones, sometimes the link is quite straightforward (e.g., in the case of ethylene responsive transcription factors upregulated in later stages). Also, mechanisms linking redox signaling and TF activity have been proposed [42]. Several DEG clusters can be pointed out, which are likely to comprise key regulatory genes, namely cDEG.1.2.11, cDEG.1.2.19, and cDEG.1.2.27 (young plant vs. immature root) and cDEG.1.3.18, cDEG.1.3.19, and cDEG.1.3.34 (young plant vs. mature root), which can be indicated as essential to understand the storage root formation. They comprise most of the differentially expressed TFs and genes involved in post-translational protein modifications together with genes directly responsible for developmental processes.

#### 3.3.2. DEGs Involved in the Carrot Storage Root Development

Plant growth and organ development is closely related to the regulation of the cell cycle. Cyclins and cyclin-dependent kinases, controlled by hormones, (de)phosphorylation, and ubiquitination followed by proteolysis are the main components of cell cycle governance [43,44]. Callose synthase is responsible for cell plate formation during cytokinesis [45], whereas microtubule array genes (e.g., MAP-65) are involved in many key processes in plant cell morphogenesis, including cell division and expansion [46]. We identified many genes associated with the cell cycle regulation and mitosis in both wild and cultivated carrots, most of them being upregulated in developing storage roots, in contrast to wild carrot roots. Clusters cDEG.1.2.11, cDEG.1.2.19, and cDEG.1.2.31 comprised most of these genes in the comparison between young plants and developing roots, while clusters cDEG.1.3.18 and cDEG.1.3.34 were enriched in this category when mature roots were compared to young plants. Cell cycle-associated genes were mostly downregulated in mature roots, as compared with developing roots (cDEG.2.3.12). Overall, it points to the importance of cell cycle regulation for the secondary growth of carrot storage roots. Beside cell cycle control, the abovementioned clusters were also associated with root and vascular strand development, cell wall biogenesis, and maintenance of the vegetative phase. Moreover, their co-expression with transcription regulators, genes involved in hormones signaling or ubiquitination, indicates multi-directional regulation and intricacy of those processes.

Another feature differentiating cultivated roots from wild carrot is the ability to uptake and store large amounts of water. Water uptake and flow through the plants roots is mainly regulated by aquaporins—proteins forming water-selective channels—facilitating water flow across membranes [47]. Differential expression of aquaporin encoding genes between wild and cultivated carrots has been previously reported by Rong et al. (2014) [7]. Aquaporin genes were upregulated in all comparisons (cDEG.1.2, cDEG.1.3, and cDEG.2.3) but not differentially expressed in wild carrot roots. Moreover, gene encoding aquaporin TIP2-2 (LOC108206639) was one of the highly upregulated genes during the storage root development (FCh >10 in cDEG.2.3). It was also revealed as differentially expressed in the direct comparison between the wild and the cultivated plants in the mature roots (wcDEG.3, Table S24 in the Supplementary Materials). Our results support the hypothesis proposed by Rong et al. (2014) that transcriptional regulation of aquaporin genes was under selection upon carrot domestication [7]. Besides aquaporins, we also identified another DEG (epidermis-specific secreted glycoprotein EP1—LOC108223777) previously reported as linked to water transport in carrots [48], highly expressed in the developing storage root as related to the young plant (cDEG.1.2, Table S14 in the Supplementary Materials), but also upregulated in immature storage roots, as compared to wild roots (wcDEG.2, Table S23 in the Supplementary Materials).

In the developing carrot storage root, sucrose is the major transport and storage sugar, but it is partially converted into starch [49,50]. Starch synthesis probably maximizes sugar gradient to enhance sink activity. During root growth and after harvest, starch and sugar concentration fluctuate, not only due to starch synthesis but also degradation [50]. In this work we identified DEGs associated with both sugars and starch metabolism. The great majority of them were upregulated in carrot storage roots but not in wild roots, indicating that carbohydrate accumulation and metabolism differentiate the two types. DEGs associated with carbohydrate metabolism were assigned to multiple clusters. Carbohydrates may also play a role in other biological processes (e.g., signal transduction [51]).

One of the key features of cultivated carrots is their ability to accumulate large amounts of carotenoids in roots. We found that two constitutive genes from the carotenoid pathway (i.e., *PSY2* and *LCYE*) were downregulated in mature roots of the cultivated carrot (cDEG.2.3). This is in line with observations that carotenoids are mostly produced in developing storage roots. Other chloroplast genes associated with photosynthesis were co-expressed with these carotenoid genes (e.g., 28 kDa ribonucleoprotein, chloroplastic—LOC108202773; chlorophyll a-b binding protein—LOC108210794; photosystem I reaction center subunit psaK—LOC108209242; photosystem II reaction center PSB28 protein—LOC108204268; ribulose bisphosphate carboxylase small chain 1B—LOC108208532; and thylakoid lumenal 29 kDa protein—LOC108219534). Generally, genes encoding proteins involved in photosynthesis were differentially expressed and highly enriched during storage root development. Moreover, three of the abovementioned genes (LOC108202773, LOC108208532, and LOC108219534) were expressed in developing storage roots but repressed in developing wild carrot roots (wcDEG.2, Table S23 in the Supplementary Materials). Enhanced expression of genes encoding proteins of photosystem II (LHC-II), in the cultivated carrot roots has been previously reported [7,52]. The authors suggested that the high expression of LHC-II genes might be related to carotenoid accumulation. Recently, this hypothesis was supported and extended by Iorizzo et al. (2016) [9]. They found that genes involved in the assembly and function of photosystems I and II and in plastid development were co-expressed with isoprenoid pathway genes (responsible for carotenoid biosynthesis) in the orange-rooted carrot. They hypothesized that loss of the cross-talk repression mechanism between the carotenoid biosynthesis pathway and the photosystems in the root tissue conditioned by inactivation of the *Y* gene induced a constitutive activation of the metabolic cascade leading to carotenoid accumulation [9]. Our results clearly corroborate that hypothesis.

## 4. Conclusions

Using transcriptomics evidence, we provided an in-depth view into the complexity of processes leading to the formation of the carrot storage root. Several interconnected regulatory and signaling mechanisms are likely involved in the storage root development. A range of differentially regulated genes encoding transcription factors and proteins involved in post-translational protein modifications have been revealed. In contrast, genes encoding proteins involved in redox signaling were largely downregulated in cultivated carrot roots, as opposed to wild carrots. Genes encoding components of photosystems I and II and those required for carotenoid biosynthesis were downregulated only in mature roots of cultivated carrots. Genes associated with cell cycle regulation were upregulated in the roots of cultivated carrot and co-expressed with genes involved in vascular strand development, likely playing a key role in the dynamics of the secondary root growth. Aquaporins were highly upregulated in all stages of carrot storage root development, possibly facilitating water uptake, but also transport of signaling molecules. The upregulation of genes encoding proteins involved in sugar metabolism was another hallmark feature of the cultivated carrot storage root. The reported results provide directions for future investigations on the regulation of storage root formation in carrots and possibly can be extended to other root crops from the Apiaceae family.

## Figures and Tables

**Table 1:** Description of the method used to label the reported comparisons and DEG clusters. GO—gene ontology.

Type of Comparison		Time Points		Cluster Number		Codes Used in the Text
Code	Description	Code	Description	Code	Description	
cDEG.	‘c’ refers to DEGs observed in the cultivated roots but not in the wild roots	1.2.	Digits refer to the timepoints (T1 vs. T2, T1 vs. T3 or T2 vs. T3) used for comparison	XX	Consecutive numbering resulting from GO enrichment analysis; ‘XX’ stands for the one or two digits representing the clusters	cDEG.1.2.XX
		1.3.				cDEG.1.3.XX
		2.3.				cDEG.2.3.XX
wDEG.	‘w’ refers to DEGs observed in the wild roots but not in the cultivated roots	1.2.				wDEG.1.2.XX
		1.3.				wDEG.1.3.XX
		2.3.				wDEG.2.3.XX
wcDEG.	‘wc’ refers to DEGs observed for wild vs. cultivated comparison	1	Digit refers to the timepoint (T1, T2, or T3) for which the wild and cultivated transcriptomes were compared			wcDEG.1.XX
		2				wcDEG.2.XX
		3				wcDEG.3.XX

**Table 2:** Transcription factors differentially expressed in the cultivated carrot.

Comparison	Cluster ID	Gene ID	Functional Annotation	log2FoldChange
cDEG.1.2	11	LOC108218660	transcription factor GTE4-like	2.218323
cDEG.1.2	11	LOC108218615	transcription factor TCP8-like	2.115051
cDEG.1.2	11	LOC108227595	transcription factor GTE8-like	2.110012
cDEG.1.2	11	LOC108204589	trihelix transcription factor GT-2-like	1.567139
cDEG.1.2	11	LOC108208638	trihelix transcription factor ASIL1-like	1.502276
cDEG.1.2	11	LOC108205163	transcription factor EGL1-like	1.268485
cDEG.1.2	19	LOC108196018	transcription factor GTE7-like	1.534413
cDEG.1.2	19	LOC108215981	transcription factor IIIB 90 kDa subunit-like	1.434734
cDEG.1.2	19	LOC108219662	GATA transcription factor 11-like	1.210731
cDEG.1.2	19	LOC108205675	transcription factor TCP8-like	1.074681
cDEG.1.2	20	LOC108196925	transcription factor MYB1R1-like	1.705882
cDEG.1.2	27	LOC108219074	trihelix transcription factor PTL	2.193835
cDEG.1.2	27	LOC108209692	ethylene-responsive transcription factor ERF118-like	2.100109
cDEG.1.2	27	LOC108209999	transcription factor MYB1R1-like	1.675776
cDEG.1.2	27	LOC108200607	GATA transcription factor 16	1.372083
cDEG.1.2	27	LOC108219053	ethylene-responsive transcription factor RAP2-13	1.068196
cDEG.1.2	31	LOC108223963	bZIP transcription factor 60-like	1.437431
cDEG.1.2	31	LOC108219044	transcription factor TCP20	1.274651
cDEG.1.2	31	LOC108218833	bZIP transcription factor 17-like	1.194341
cDEG.1.3	18	LOC108204549	general transcription factor 3C polypeptide 3	2.767626
cDEG.1.3	18	LOC108208932	heat stress transcription factor C-1-like	2.14659
cDEG.1.3	18	LOC108204669	helicase-like transcription factor CHR28	1.576643
cDEG.1.3	18	LOC108219601	WRKY transcription factor 1-like	1.371966
cDEG.1.3	18	LOC108205270	GATA transcription factor 26-like	1.267808
cDEG.1.3	18	LOC108227355	transcription factor bHLH130-like	1.150305
cDEG.1.3	19	LOC108218615	transcription factor TCP8-like	3.326214
cDEG.1.3	19	LOC108218436	nuclear transcription factor Y subunit A-10	2.583937
cDEG.1.3	19	LOC108205657	WRKY transcription factor 21	2.093602
cDEG.1.3	19	LOC108213173	ethylene-responsive transcription factor RAP2-1-like	1.936345
cDEG.1.3	19	LOC108210769	WRKY transcription factor 69	1.581155
cDEG.1.3	19	LOC108215331	WRKY transcription factor 21	1.340796
cDEG.1.3	19	LOC108205942	transcription factor MYB1R1-like	1.16443
cDEG.1.3	19	LOC108213035	transcription factor bHLH68	0.964932
cDEG.1.3	19	LOC108203417	transcription factor GTE2-like	0.924269
cDEG.1.3	19	LOC108219684	trihelix transcription factor ASIL2	0.814925
cDEG.1.3	28	LOC108200607	GATA transcription factor 16	1.452332
cDEG.1.3	28	LOC108196925	transcription factor MYB1R1-like	1.179838
cDEG.1.3	34	LOC108219539	transcription factor TGA1-like	2.492909
cDEG.1.3	34	LOC108200279	WRKY transcription factor 57	1.972727
cDEG.1.3	34	LOC108227595	transcription factor GTE8-like	1.901753
cDEG.1.3	34	LOC108223963	bZIP transcription factor 60-like	1.775269
cDEG.1.3	34	LOC108206522	ethylene-responsive transcription factor 4-like	1.713784
cDEG.1.3	34	LOC108214474	transcription factor LHW-like	1.695994
cDEG.1.3	34	LOC108197784	WRKY transcription factor 28	1.669975
cDEG.1.3	34	LOC108208638	trihelix transcription factor ASIL1-like	1.472099
cDEG.1.3	34	LOC108218767	WRKY transcription factor 3	1.434899
cDEG.1.3	34	LOC108194726	trihelix transcription factor ASR3	1.425662
cDEG.1.3	34	LOC108227612	transcription factor 25	1.400666
cDEG.1.3	34	LOC108194205	ethylene-responsive transcription factor ERF008-like	1.361216
cDEG.1.3	34	LOC108197411	transcription factor bHLH113-like	1.250711
cDEG.1.3	34	LOC108205979	trihelix transcription factor GT-1	1.052866
cDEG.2.3	3	LOC108220123	ethylene-responsive transcription factor 2-like	1.291574
cDEG.2.3	5	LOC108211036	transcription factor PCL1-like	−1.98882
cDEG.2.3	5	LOC108224748	nuclear transcription factor Y subunit C-1-like	−1.72038
cDEG.2.3	5	LOC108218926	NAC transcription factor 29-like	−1.58027
cDEG.2.3	12	LOC108220417	transcription factor MYB48-like	−2.28333
cDEG.2.3	12	LOC108214397	heat stress transcription factor A-3-like	−1.49692
cDEG.2.3	14	LOC108211325	ethylene-responsive transcription factor ERF010-like	2.266422
cDEG.2.3	14	LOC108197506	transcription factor bHLH147	1.218184
cDEG.2.3	14	LOC108192438	nuclear transcription factor Y subunit A-1-like	1.217534

## Supplementary Materials

The Supplementary Material for this article can be found online at http://www.mdpi.com/2073-4425/9/9/431/s1, including the following: Figure S1: Venn diagrams representing numbers of DEGs identified with three different algorithms for the wild *D. carota* subsp. *commutatus*; Figure S2: Venn diagrams representing numbers of DEGs identified with three different algorithms for the cultivated *D. carota* subsp. *sativus* 2874B; Figure S3: Venn diagrams representing numbers of DEGs identified with three different algorithms for the comparison between the wild *D. carota* subsp. *commutatus* and the cultivated *D. carota* subsp. *sativus* 2874B, and Tables S1–S26. The RNAseq datasets generated for this study can be found in the GenBank Short Read Archive acc. no. SRP155333.
