# Supplementary material for: Comparative Transcriptomics of Root Development in Wild and Cultivated Carrots
Source: Genes (Basel). 2018 Aug 24;9(9):431. doi: 10.3390/genes9090431 (PMC6162504; doi:10.3390/genes9090431)
Supplement: Supplementary file 1 [file genes-09-00431-s001.zip › Supplementary Figures S1-S3.pdf]

S1

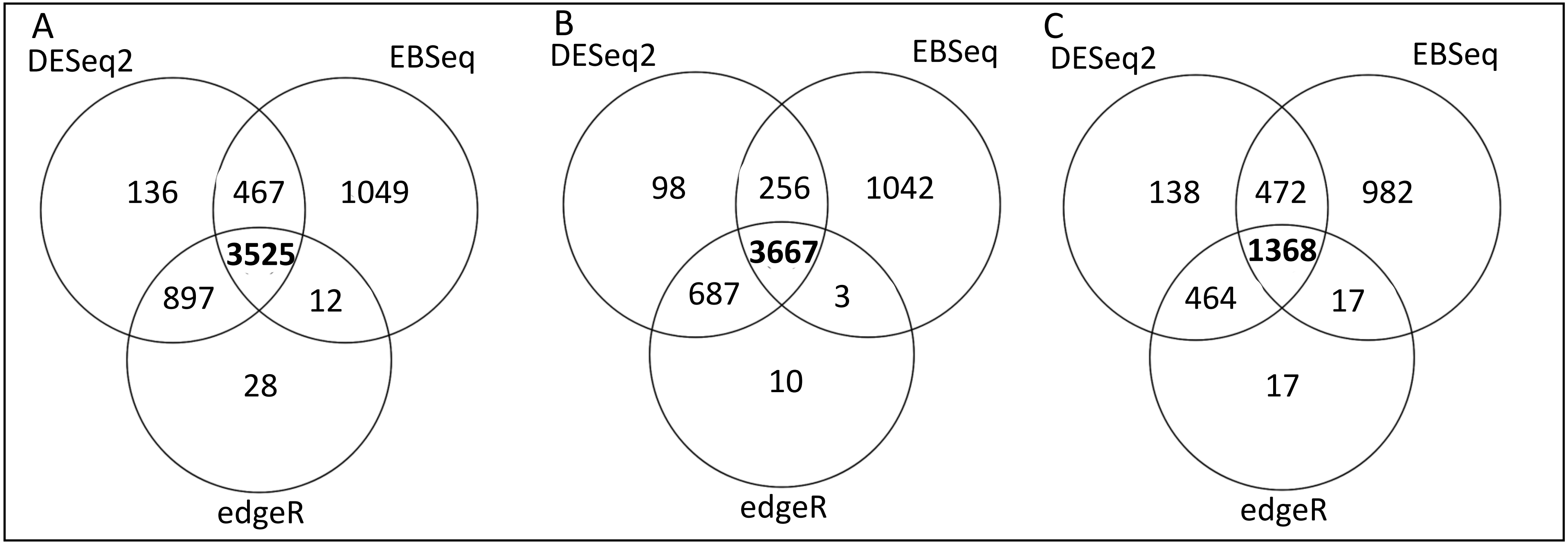

Figure S1. Venn diagrams representing numbers of DEGs identified with three different algorithms for the wild *D. carota* subsp. *commutatus* in comparisons between time points T1 vs. T2 – wDEG.1.2 (A), T1 vs. T3 – wDEG.1.3 (B), and T2 vs. T3 – wDEG2.3 (C). Only DEGs confirmed by all three methods were used for subsequent analyses.

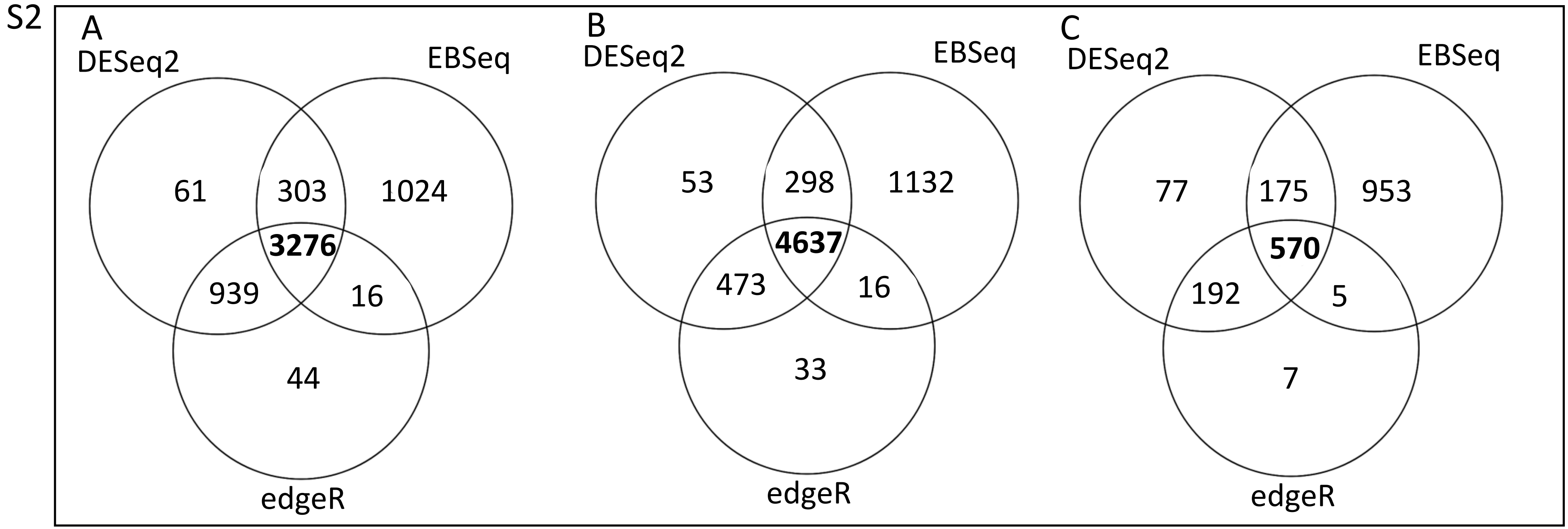

Figure S2. Venn diagrams representing numbers of DEGs identified with three different algorithms for the cultivated *D. carota* subsp. *sativus* 2874B in comparisons between time points T1 vs. T2 – cDEG.1.2 (A), T1 vs. T3 – cDEG.1.3 (B), and T2 vs. T3 – cDEG2.3 (C). Only DEGs confirmed by all three methods were used for subsequent analyses.

S3

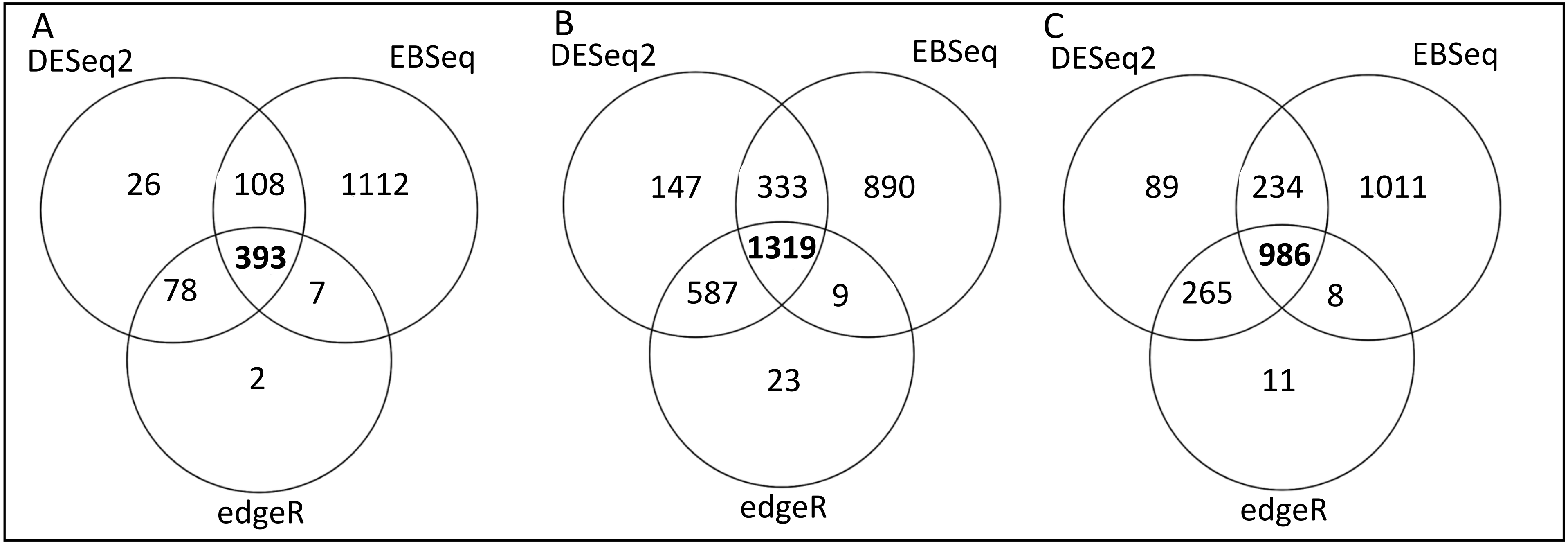

Figure S3. Venn diagrams representing numbers of DEGs identified with three different algorithms for the comparison between the wild *D. carota* subsp. *commutatus* and the cultivated *D. carota* subsp. *sativus* 2874B in time point T1– wcDEG.1 (A), T2 – wcDEG.2 (B), and T3 – wcDEG.3 (C). Only DEGs confirmed by all three methods were used for subsequent analyses.
